# High sensitivity hydrogen analysis in zircaloy-4 using helium-assisted excitation laser-induced breakdown spectroscopy

**DOI:** 10.1038/s41598-021-01601-y

**Published:** 2021-11-09

**Authors:** Marincan Pardede, Indra Karnadi, Rinda Hedwig, Ivan Tanra, Javed Iqbal, Mangasi Alion Marpaung, Maria Margaretha Suliyanti, Eric Jobiliong, Syahrun Nur Abdulmadjid, Nasrullah Idris, Ali Khumaeni, Muhandis Shiddiq, Mario Gracio Anduinta Rhizma, Zener Sukra Lie, Muhammad Bilal, Davy Putra Kurniawan, Tjung Jie Lie, Koo Hendrik Kurniawan, Kiichiro Kagawa

**Affiliations:** 1 Department of Electrical Engineering, University of Pelita Harapan, Tangerang, 15811 Indonesia; 2 Department of Electrical Engineering, Krida Wacana Christian University, Jakarta, 11470 Indonesia; 3 Computer Engineering Department, Faculty of Engineering, Bina Nusantara University, Jakarta, 11480 Indonesia; 4 Department of Physics, University of Azad, Jammu & Kashmir, Muzaffarabad, Pakistan; 5 Faculty of Mathematics and Natural Sciences, Jakarta State University, Jakarta, 13220 Indonesia; 6 Research Center for Physics, Indonesia Institute of Science, Kompleks Puspiptek, Tangerang, Selatan 15314 Indonesia; 7 Department of Industrial Engineering, University of Pelita Harapan, Tangerang, 15811 Indonesia; 8 Department of Physics, Faculty of Mathematics and Natural Sciences, Syiah Kuala University, Darussalam, Banda Aceh, 23111 Indonesia; 9 Department of Physics, Faculty of Mathematics and Natural Sciences, Diponegoro University, Semarang, 50275 Indonesia; 10 Automotive & Robotics Program, Computer Engineering Department, Binus ASO School of Engineering, Bina Nusantara University, Jakarta, 11480 Indonesia; 11 Laser Matter Interaction Laboratory, Department of Physics, Faculty of Sciences, University of Agriculture Faisalabad, Faisalabad, 38040 Pakistan; 12 Research Center of Maju Makmur Mandiri Foundation, 40/80 Srengseng Raya, Jakarta, 11630 Indonesia; 13 Fukui Science Education Academy, Takagi Chuou 2 Choume, Fukui, 910-0804 Japan

**Keywords:** Physics, Plasma physics, Laser-produced plasmas

## Abstract

High-sensitivity detection of hydrogen (H) contained in zircaloy-4, a commonly used material for nuclear fuel containers, is crucial in a nuclear power plant. Currently, H detection is performed via gas chromatography, which is an offline and destructive method. In this study, we developed a technique based on metastable excited-state He-assisted excitation to achieve excellent quality of H emission spectra in double-pulse orthogonal laser-induced breakdown spectroscopy (LIBS). The production of metastable excited-state He atoms is optimized by using LiF as sub-target material. The results show a narrow full-width-at-half-maximum of 0.5 Å for the H I 656.2 nm emission line, with a detection limit as low as 0.51 mg/kg. Thus, using this novel online method, H in zircaloy-4 can be detected efficiently, even at very low concentrations.

## Introduction

The advantages offered by nuclear power are unmatched with those provided by other alternative energy sources, in terms of satisfying the ever-growing energy demands of the modern world. Although some accidents have occurred in the past, several new nuclear power plants are still being built and planned. These new power plants generally employ improved safety control measures and operational procedures. Zircaloy tubes of approximately 10-mm diameter are used as radioactive fuel containers in nuclear power plants. During operation of a nuclear power plant, hot water reacts with the surface of these zircaloy tubes and produces zirconium oxide along with hydrogen gas, which tends to penetrate the zircaloy tubes. Over time, the H concentration in the zircaloy tubes increase. This causes structural damage and reduces the strength of the zircaloy tubes considerably. Therefore, the amount of H in the tubes should be routinely inspected. Currently, H trapped in zircaloy tubes is detected using gas detectors. However, this technique is highly destructive, dangerous, time-consuming, labor-intensive, and expensive because it requires the power plant operation to be halted and the radioactive material must be extracted from the zircaloy tube. A part of the zircaloy tube is then melted in a carbon furnace before performing gas chromatography^1,2^.

Laser-induced breakdown spectroscopy (LIBS) is widely recognized as a highly efficient and rapid spectrochemical analysis method for a large variety of material samples. The recent successful geological explorations on Mars demonstrated the potential applicability of LIBS in spectrochemical analysis^3–8^. However, despite its wide applicability, detection of very light elements, such as H, using LIBS is still challenging. In our previous studies on H analysis, starting from 2004^9–16^, we observed that the inability of LIBS to detect H is mainly due to two reasons: First, a very high-density plasma, naturally containing a large amount of charged particles, is produced in LIBS. These charged particles produce a strong internal field, thereby causing an extreme Stark broadening effect, particularly for the H emission lines. Second, the emission intensity of the H emission lines decreases rapidly with increasing gas pressure and becomes undetectable at 1 atmospheric pressure. This was explained within the framework of the shock wave excitation model in terms of a mismatch between the fast passage of the relatively light H atoms and the formation of the shock wave, which is mainly induced by the heavier atoms of the host target. As a result of the mismatch, the H atoms largely miss the shock wave, which is responsible for their excitations. At a higher surrounding gas pressure, the starting of the shock wave is delayed even further, leading to a greater mismatch between the two processes. This explains the inapplicability of LIBS in H analysis on solid samples.

Recently, we used metastable excited-state He atoms (He*) as excitation sources for ablated H atoms in a Penning-like collision process. We used double-pulse (DP) orthogonal LIBS with He ambient gas. This requires a two-laser system in which the first laser is used to generate the He gas plasma and the second laser is used to ablate the target. The time synchronization between the two lasers systems is adjusted such that the ablated H atoms from the target enter the relatively cool He gas plasma in which the ions and electrons have already recombined, yielding a very low background and a very sharp H emission line^17–35^. This technique involves using a DP orthogonal system, which is completely different from ordinary DP systems. In ordinary DP systems, the first laser is used to ablate the target and the second laser, with a certain delay, is used to excite or re-excite the ablated atoms^36–38^. Despite the reasonably high H spectral quality obtained in previous experiments, the He gas plasma, which exhibited a typical orange color as a result of the He I 587.6 nm emission, suffered from instability when generated using atmospheric-pressure ambient He gas. To overcome this problem, we changed the surrounding He gas pressure from atmospheric pressure to a low pressure of 3 kPa. Under this condition, H and D analysis could be performed with a detection limit of 10 mg/kg^39,40^.

To increase the detection limit of H in zircaloy pipes, H emission must be maximized. Since the number of H atoms in zircaloy samples are fixed and since H is excited through a Penning-like energy transfer from He* atoms, the energy transfer efficiency must be increased. The transfer efficiency can be increased only if a large number of He* atoms are produced in the He gas plasma. He gas plasma can only be formed at a low pressure if the laser beam is focused and hits a certain sub-target. The intensities of He I 587.6 nm and He I 667.8 nm, which are related to the He* atoms, change depending on the type of sub-target^1,2,23–29^. In this study, we varied the sub-target material from metals to inorganic compounds and found that LiF yields He I 587.6 nm and He I 667.8 nm emission lines with the maximum intensity. Spectrochemical analysis of H in zircaloy-4 was performed using LiF as a sub-target. The results showed that the detection limit of H in zircaloy could be as low as 0.51 mg/kg, which is 20 times better than the previously reported limit^40^.

## Materials and methods

**Figure 1:**
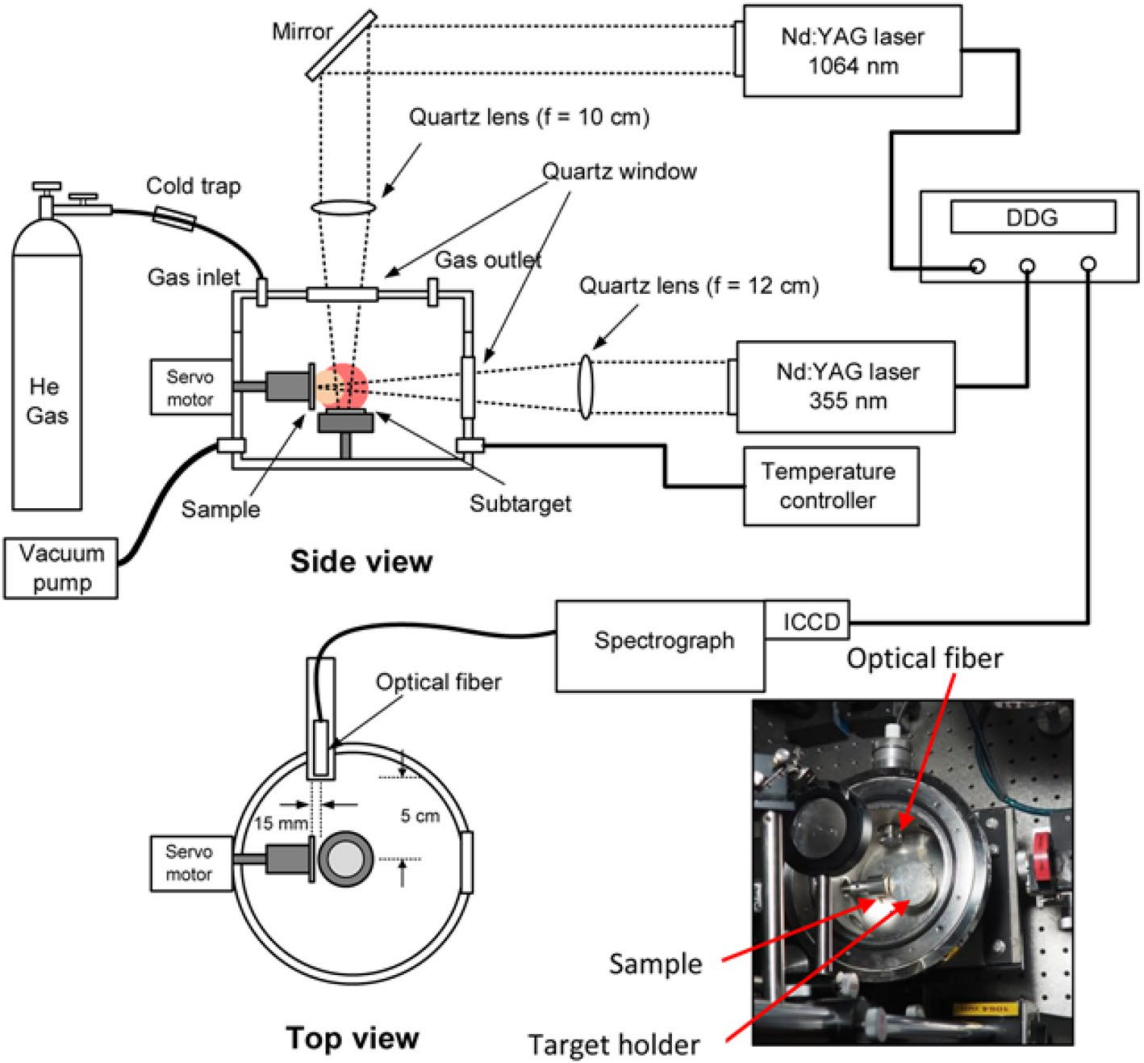
Schematic of experimental setup.

A schematic diagram of the experimental setup used in this study is shown in Fig. 1. The system consisted of two Nd:YAG nanosecond (ns) lasers, a high-resolution spectrograph, a digital delay generator, and a vacuum chamber. The first laser (Quanta Ray, Lab 130-10, 1064 nm, 450 mJ) was operated in the Q-switched mode with a 10-Hz repetition rate. Its energy was reduced and fixed at 82 mJ. This energy is selected so that it can be achieved even using a small compact laser, which is preferable for in situ zircaloy-4 analysis in a nuclear power plant. The first laser used in this study was focused onto the sub-target surface using a plano-convex quartz lens of focal length 100 mm under a defocused position of − 5 mm, such that the real distance between the surfaces of the lens and sub-target was 95 mm. This adjustment was performed to avoid excessive sub-target ablation during the laser irradiation. We also found that the intensity of the He line emissions were almost the same, even when the laser beam was tightly focused. We estimated the power density under the defocused condition to be around 0.4 GW/cm^2^. The generated He gas plasma had a diameter of approximately 30 mm and exhibited an intense orange color representing the He I 587.6 nm and He I 667.8 nm emission lines. The second laser (Quanta Ray, INDI 10, 355 nm, 100 mJ) was also operated in the Q-switched mode with the same 10-Hz repetition rate. However, its energy was reduced and fixed at 11 mJ. This laser was tightly focused onto the zircaloy-4 sample using a plano-convex lens with a focal length of 12 cm, yielding a power density of approximately 1 GW/cm^2^. The generated zircaloy-4 plasma had a diameter of approximately 25 mm. The laser energy was reduced to around 11 mJ to minimize the surface damage to the zircaloy-4 sample. Synchronization between the two laser systems was performed via a digital delay generator (Stanford Research System, DG 535).

The light emitted from the two resulting plasmas was collected using an optical fiber that was placed at 5 cm alongside of the plasmas, as shown in Fig. 1. Under the aforementioned condition, the fiber could collect the emission of both plasmas from the entire region. The detection system consisted of a spectrograph (McPherson, model 2061, f = 1000 mm, Czerny Turner configuration) and a gated intensified charge-coupled device (ICCD, Andor 256 × 1024 pixels). The gate delay and gate width of the ICCD were fixed at 200 ns and 50 µs, respectively, in accordance with the operation of the second laser, to yield the most favorable emission spectra^1,2,39,40^.

A special cylindrical metal chamber with a 115-mm inner diameter and several entry ports was designed and constructed. The main targets that were used in this experiment consisted of a set of zircaloy-4 samples doped with different concentrations of H (150 mg/kg, 400 mg/kg, and 800 mg/kg). All the samples had a 10 mm × 10 mm cross-sectional area and a 1-mm thickness. The sample holder was designed to accommodate four samples simultaneously. The sub-target sample holder was placed perpendicular to the sample holder, as depicted in the inset of Fig. 1, which shows a top-view image of the experimental chamber. After placing the sample and sub-target inside the chamber, the chamber was evacuated to a pressure of 1 Pa using a vacuum pump. Next, the chamber was heated to 473 K for 30 min to remove most of the surface water, before the high-purity He gas (Air Liquid, 5 N) was introduced into the chamber until a desired pressure of 3 kPa was reached. The temperature of 473 K is chosen because when it is raised more than 500 K, the trapped H atom in the zircaloy plate will evaporate. Meanwhile, based on our previous experiment^1,2^, the He gas pressure of 3 kPa is chosen to give maximum intensity of H.

To further eliminate the possibility of contamination from moisture in the He gas, a cold trap was employed in the flow line using liquid nitrogen. The gas pressure and chamber temperature were kept constant during the experiment. For the sub-target, several materials were prepared, for example, Al, Si, Ni, Cu, Zn, Ag, W, Au, Pb, and C (all obtained from Rare Metallic Co., Japan, with 4-N purity) along with pellets of LiF and KCl having a 10-mm diameter and 2-mm thickness (powder was obtained from Wako Company, Japan, with 5-N purity). Prior to the experiment, we ensured that these sub-target materials did not contain any H impurity. The absence of H impurity in the sub-targets was further confirmed via the obtained spectra, which did not exhibit any H emission lines.

## Results and discussion

The experimental results and discussion are presented in the following subsections. In the first subsection, we discuss the best conditions to obtain higher He emission intensity (He I 587.6 nm and He I 667.8 nm) which is correspond to the higher number of He metastable excited state atoms (He*). In the second subsection, we describe the best experimental conditions necessary for high-sensitivity H analysis using He* atoms.

### Maximum production of He* atoms using different sub-targets

**Figure 2:**
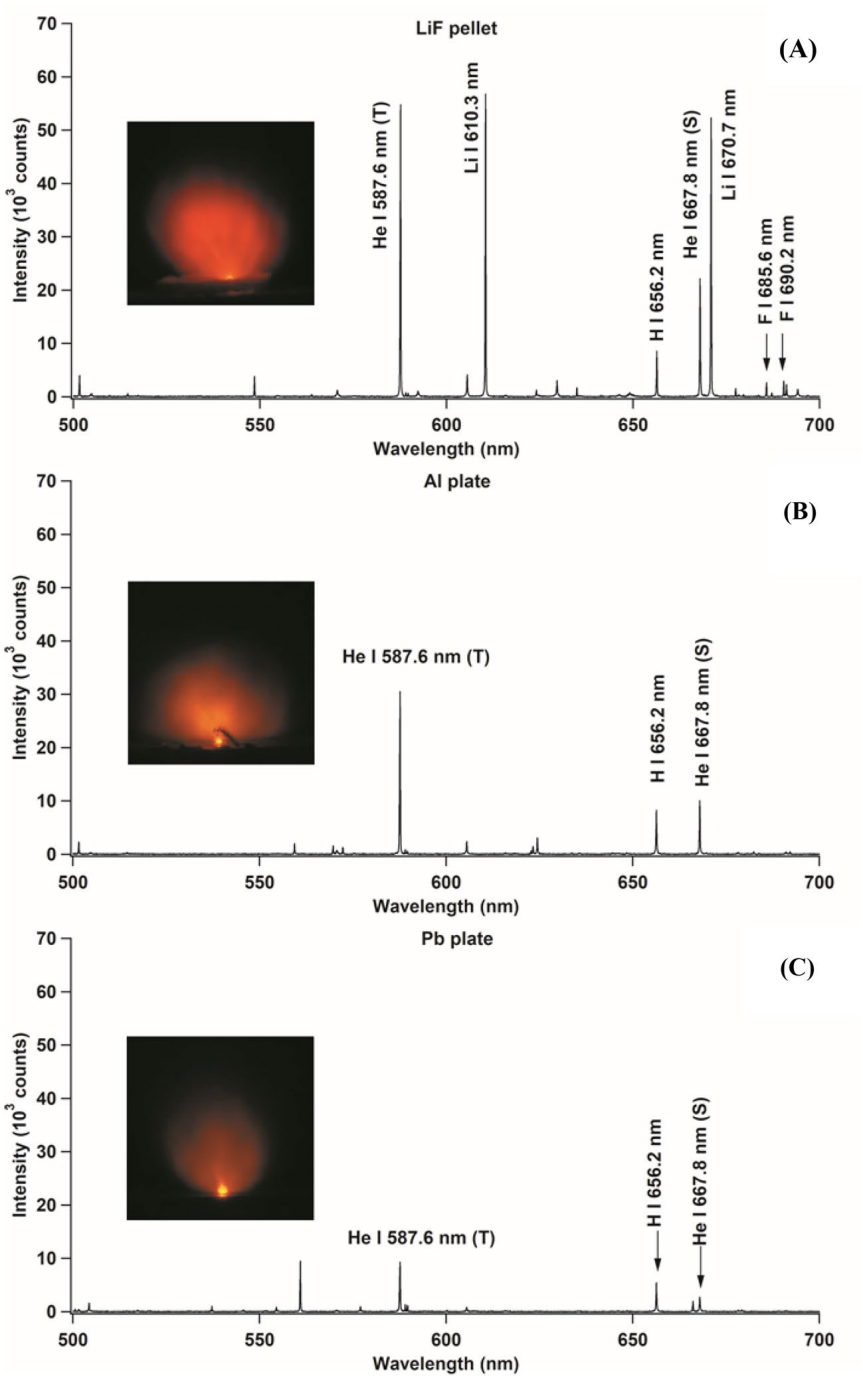
Emission spectra of the He gas plasma when only the first laser with an energy of 82 mJ is focused onto different sub-targets; (**A**) LiF, (**B**) Al, and (**C**) Pb in the surrounding He gas at 3 kPa. Inset shows a photograph of the produced plasma. The gate delay and gate width of the detection system were set as 200 ns and 50 μs, respectively, after the initiation of the first laser (*T* triplet, *S* singlet).

In this part of the investigation, we only used the first laser to generate the He gas plasma. The laser was focused on different sub-targets that were kept under a He gas atmosphere at 3 kPa inside the experimental chamber. Visual observation of the produced He gas plasma is vital because the plasma exhibits a visible orange color associated with emissions from the He I 587.6 nm (triplet) and He I 667.8 nm (singlet) lines. The inset of Fig. 2 shows the He gas plasma when the first laser of 82 mJ was focused onto the LiF, Al, and Pb sub-targets in the surrounding He gas at 3 kPa. The diameter of the produced plasma was approximately 30 mm. It is noteworthy that inferences about the emission intensities can be obtained simply through visual observation. The most intense orange color was obtained in the case of the LiF sub-target, whereas the weakest intense orange color was obtained when we used the Pb sub-target. This result exactly matched the detected emission spectra for the three cases, as shown in Fig. 2. The strongest He I 587.6 nm line was obtained for the LiF sub-target, with approximately 60,000 counts, while the counts for the Al and Pb sub-targets were around 30,000 and 9000, respectively. Note that the H I 656.2 nm emission line was observed in all the spectra. This H emission line, emitted from the surface water, was observed since we had neither heated the chamber nor used the cold trap in the He gas line for this part of the experiment.

In our previous studies^1,2,29,39,40^, we have proved that He* atoms also contribute to the excitation of He itself because He emission continues for a long time and because the excitation energy of He is considerably higher than that of other elements, including Li, Al, and Pb. It is assumed that in the He gas plasma, He* atoms are ionized because of collisions with each other via the Penning effect, as shown in Eq. (1). Subsequently, the He ions and electrons recombine to produce the higher energy state of He (He***), as shown in Eq. (2). Next, through cascade transition (Eq. (3)), He** is produced, which emits He I 587.6 nm and He I 667.8 nm (Eq. (4)).

1 $${\text{He}}^{*} \, + {\text{ He}}^{*} \, \to {\text{ He }} + \, \left( {{\text{He}}^{ + } + {\text{ e}}^{ - } } \right),$$

2 $${\text{He}}^{ + } + {\text{ e}}^{ - } \to {\text{ He}}^{***},$$

3 $${\text{He}}^{***} \, \to {\text{ He}}^{**},$$

4 $${\text{He}}^{**} \, \to {\text{ He}}^{*} \, + {\text{h}}\upupsilon.$$

**Figure 3:**
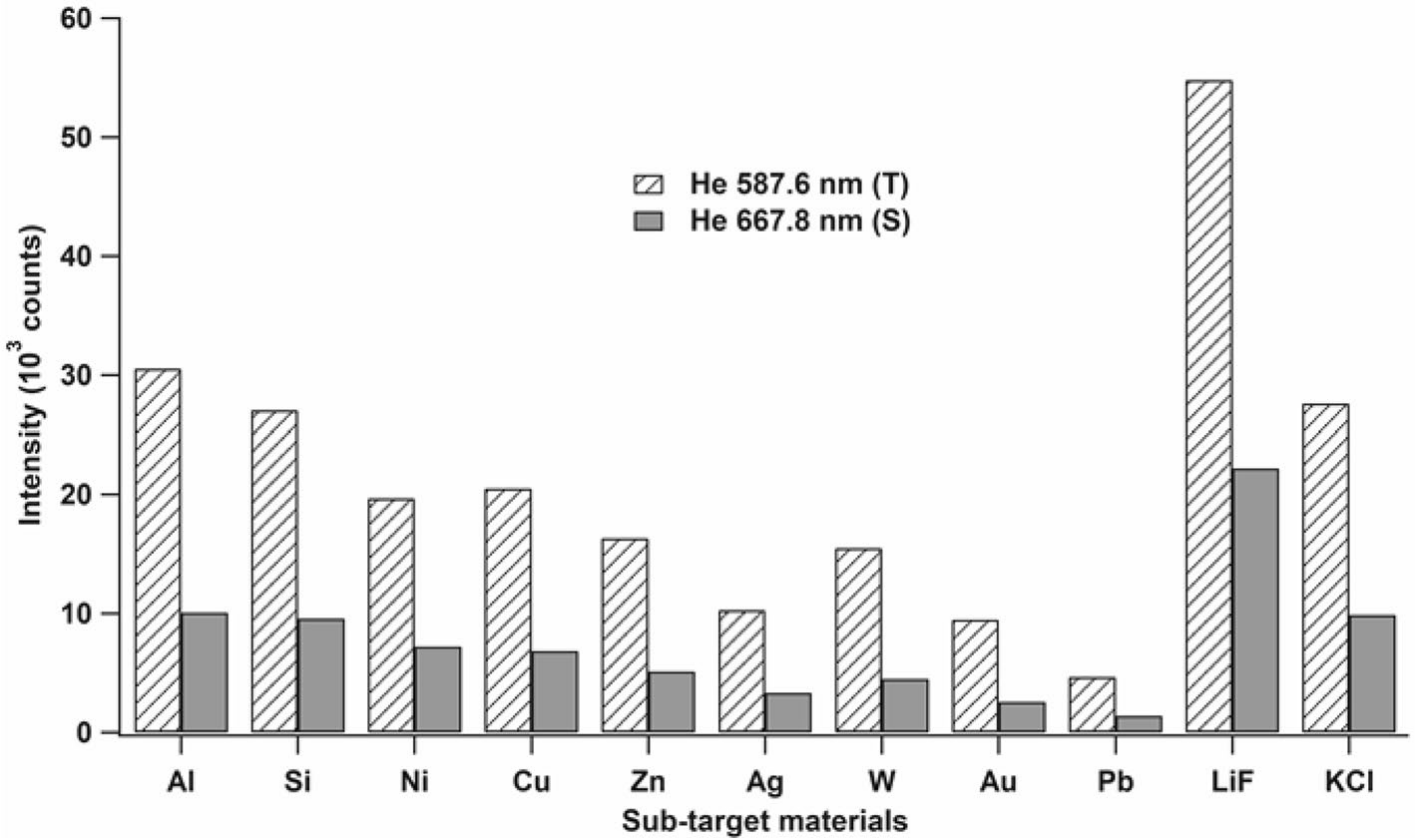
Emission intensity of He I 587.6 nm (triplet) and He I 667.8 nm (singlet) lines when only the first laser with an energy of 82 mJ is focused onto different sub-targets in the surrounding He gas at 3 kPa. The gate delay and gate width of the detection system were set as 200 ns and 50 μs, respectively, after the initiation of the first laser.

Based on this explanation, we can conclude that the higher emission intensities of He I 587.6 nm and He I 667.8 nm correspond to a higher number of He* atoms becoming readily available as excitation sources for the ablated target atoms. We also evaluated another sub-target made of a different material, such as Si, Ni, Cu, Zn, Ag, W, Au, and KCl; the corresponding emission intensities of the He I 587.6 nm (triplet) and He I 667.8 nm (singlet) lines are plotted in Fig. 3. From this figure, we can infer that the maximum emission intensity of He is obtained in the case of the LiF sub-target. Therefore, we used LiF as the sub-target for the H analysis of zircaloy-4 samples. We speculate since Li has a comparable mass with He, then these two atoms may collide elastically and the energy of the He metastable excited state atom, after and before collision is almost the same, yielding almost no reduction of the production of the He metastable excited state atoms. As a consequence, when the subtarget is Pb, which has a much larger mass, a significant reduction of the production of the He metastable excited state is observed, as clearly shown in Fig. 2.

### High-sensitivity H analysis of zircaloy-4 samples

**Figure 4:**
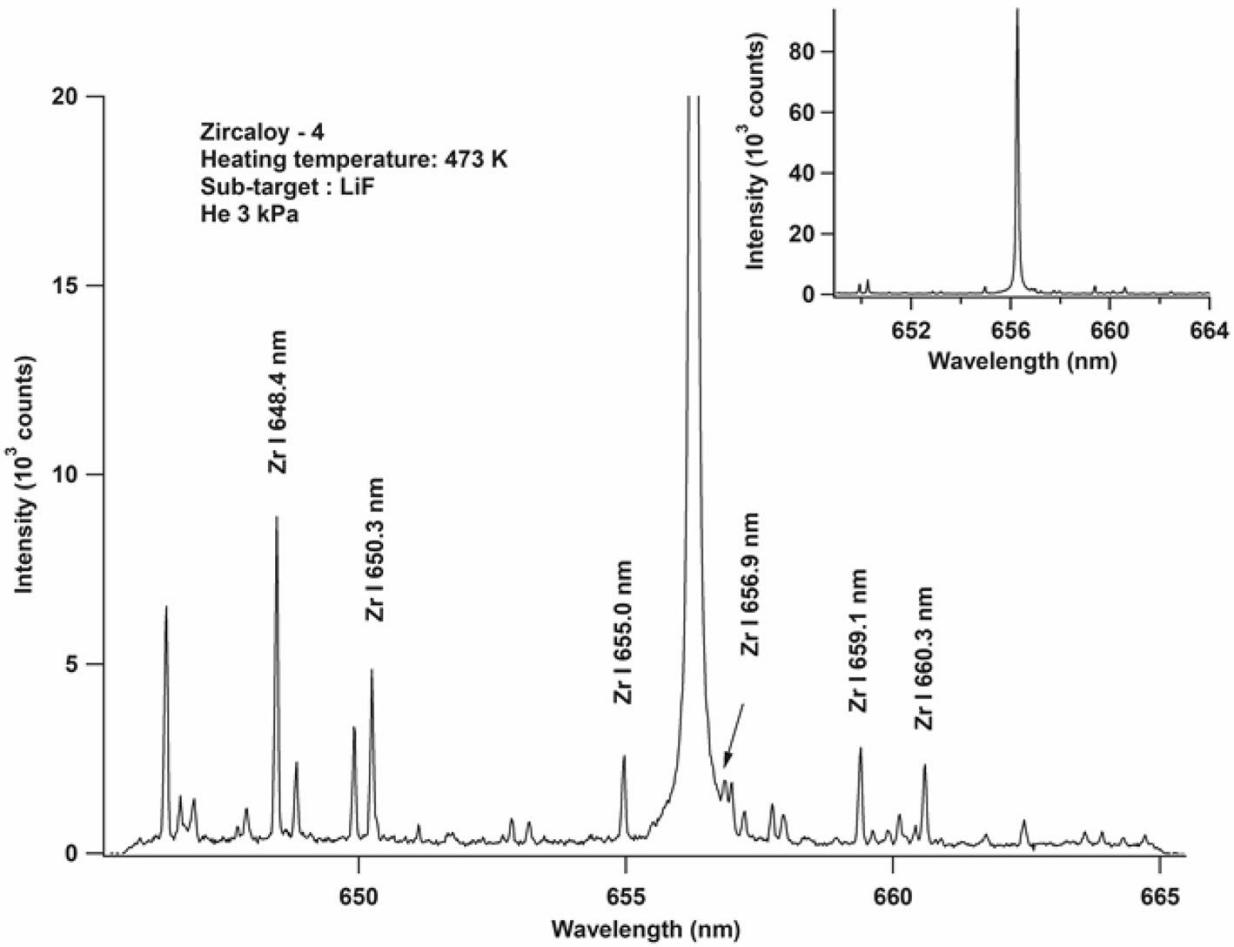
Emission spectra of zircaloy-4 sample containing 800 mg/kg of H. A double-pulse orthogonal configuration was used. The second laser was fired 0.5 μs after the first laser. The surrounding He gas pressure was kept constant at 3 kPa. The gate delay and gate width of the detection system were set as 200 ns and 50 μs, respectively, after the initiation of the second laser. Inset shows the full emission intensity of the H I 656.2 nm line.

This experiment was performed using a DP orthogonal system under a low He gas pressure of 3 kPa inside the experimental chamber, as explained in the previous sections. We focused the first laser to irradiate the LiF sub-target, and after a certain delay, the second laser was fired to ablate the zircaloy-4 sample. Through repeated measurements, it was found that a 0.5 μs delay in firing the second laser pulse indeed produced H emission spectra with the optimal quality. This delay provided the optimal window for the ablated atoms of the zircaloy-4 sample, which contained H impurity, to enter the relatively cool He plasma. This ensured that the excitation of the ablated target atoms occurred solely because of He* and not because of thermal excitation. Figure 4 shows the emission spectra of the zircaloy-4 sample, containing 800 mg/kg of H impurity. A very strong and narrow full-width-at-half-maximum (0.5 Å) was observed for the H I 656.2 nm line peak, as shown in the inset of Fig. 4. Almost no complex Zr emission lines near this wavelength (656.2 nm) were observed. However, the Zr emission lines became visible when we reduced the y-axis scale, i.e., at lower intensities, as shown in Fig. 4. Note that this experiment was conducted using a cold trap in the He gas line and heating the chamber to 473 K. Thus, we can conclude that the observed H I 656.2 nm peak originated from the H impurity in the zircaloy-4 sample, and not from a combination of H from inside the sample and surface water.

We propose that the excitation of H atoms, as shown in Fig. 4, is also due to energy transfer via the Penning-like collisions between the H atoms and He* atoms, as described in the following equations:

5 $${\text{He}}^{*} \, + {\text{ H }} \to {\text{ He }} + \, \left( {{\text{H}}^{ + } + {\text{ e}}^{ - } } \right),$$

6 $${\text{H}}^{ + } + {\text{ e}}^{ - } \to {\text{ H}}^{***},$$

7 $${\text{H}}^{***} \, \to {\text{ H}}^{**} \, \to {\text{ H}}^{*},$$

8 $${\text{H}}^{*} \, \to {\text{ H }} + {\text{h}}\upupsilon .$$

**Figure 5:**
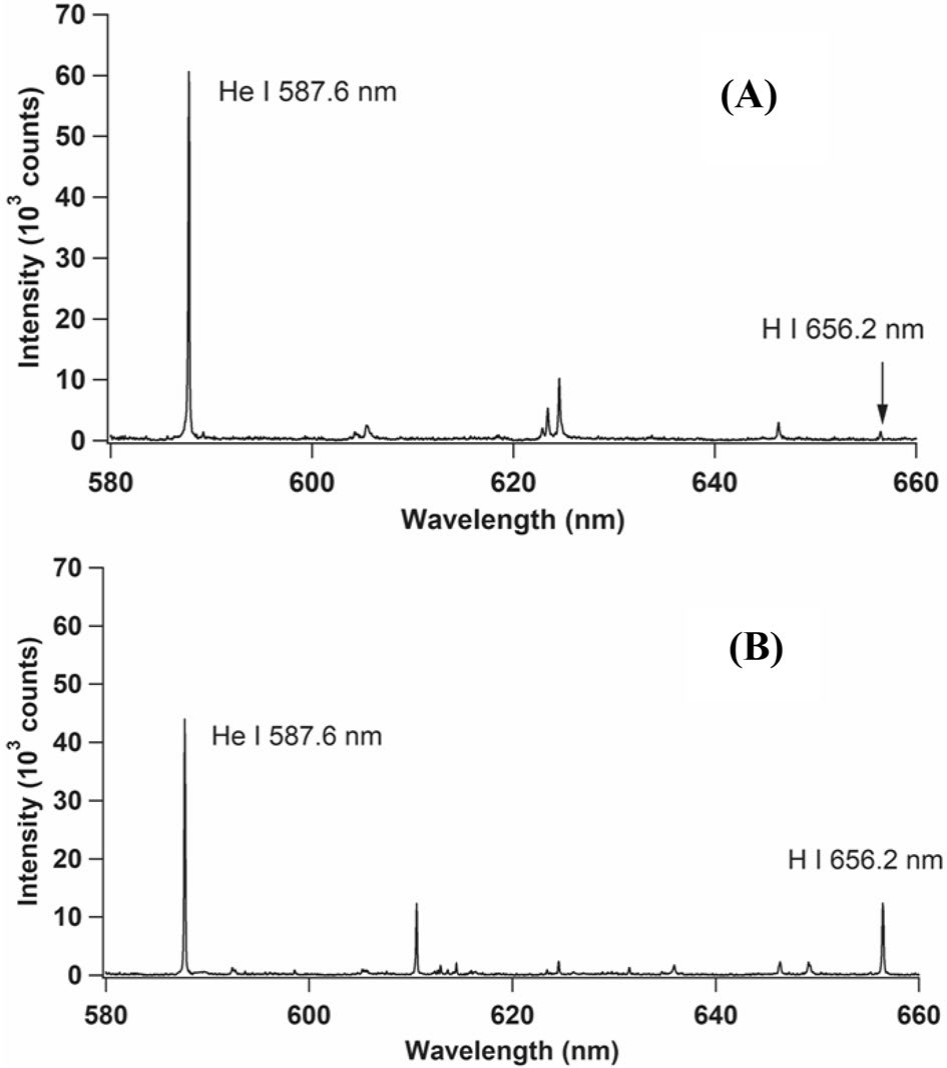
Emission spectra of zircaloy-4 sample containing 800 mg/kg of H. (**A**) Only the first laser was turned on with the same energy of 82 mJ in He gas at 3 kPa. The gate delay and gate width of the detection system were set as 200 ns and 50 μs, respectively, after the initiation of the first laser. (**B**) Both lasers were turned on as per the conditions mentioned in Fig. 4. The Echelle spectrograph of Andor M500 was used to obtain the wide wavelength range. The Al sub-target was used in this experiment.

The ablated H atoms that collide with the He* atoms are ionized, resulting in the production of highly energetic free electrons, as shown in Eq. (5). The energy of the free electron corresponds to the energy difference between the metastable excited-state energy of He and the ionization energy of the ablated atom (H). The free electrons released from H are expected to undergo multiple collisions with the H ions and recombine to produce H atoms at a higher energy state (H***), as shown in Eq. (6). This is followed by non-radiation–cascaded transitions (Eq. (7)) that produce the excitation state (H*), from which atomic emission lines of H are emitted (Eq. (8)). This scenario is exactly same as that shown in Fig. 5, which presents the emission spectra of H I 656.2 nm together with those of He I 587.6 nm when only the first laser is turned on (Fig. 5A) and when both lasers are turned on (Fig. 5B). Figure 5A shows the strong emission line of He I 587.6 nm with 60,000 counts and an extremely small H I 656.2 nm emission line, which resulted from the surface water (since we did not use a cold trap or heat the chamber). Note that this experiment is carried out using Al subtarget since water are easily trapped on the metal surface and we intended to get small H emission from surface water. As seen in Fig. 5B, the emission intensity of He I 587.6 nm decreases to nearly 67% while that of H I 656.2 nm increases by approximately 12 times when the two lasers is operated. This indicates that only a fraction of the He* atoms is used to excite the H atoms, as explained in Eqs. (5)–(8). It is also possible that a fraction of the He* atoms was used to excite Zr, which is the primary element in zircaloy-4. However, the probability of this is extremely low since the difference between the excitation energies of Zr and He* is quite large as compared with that between H and He*.

**Figure 6:**
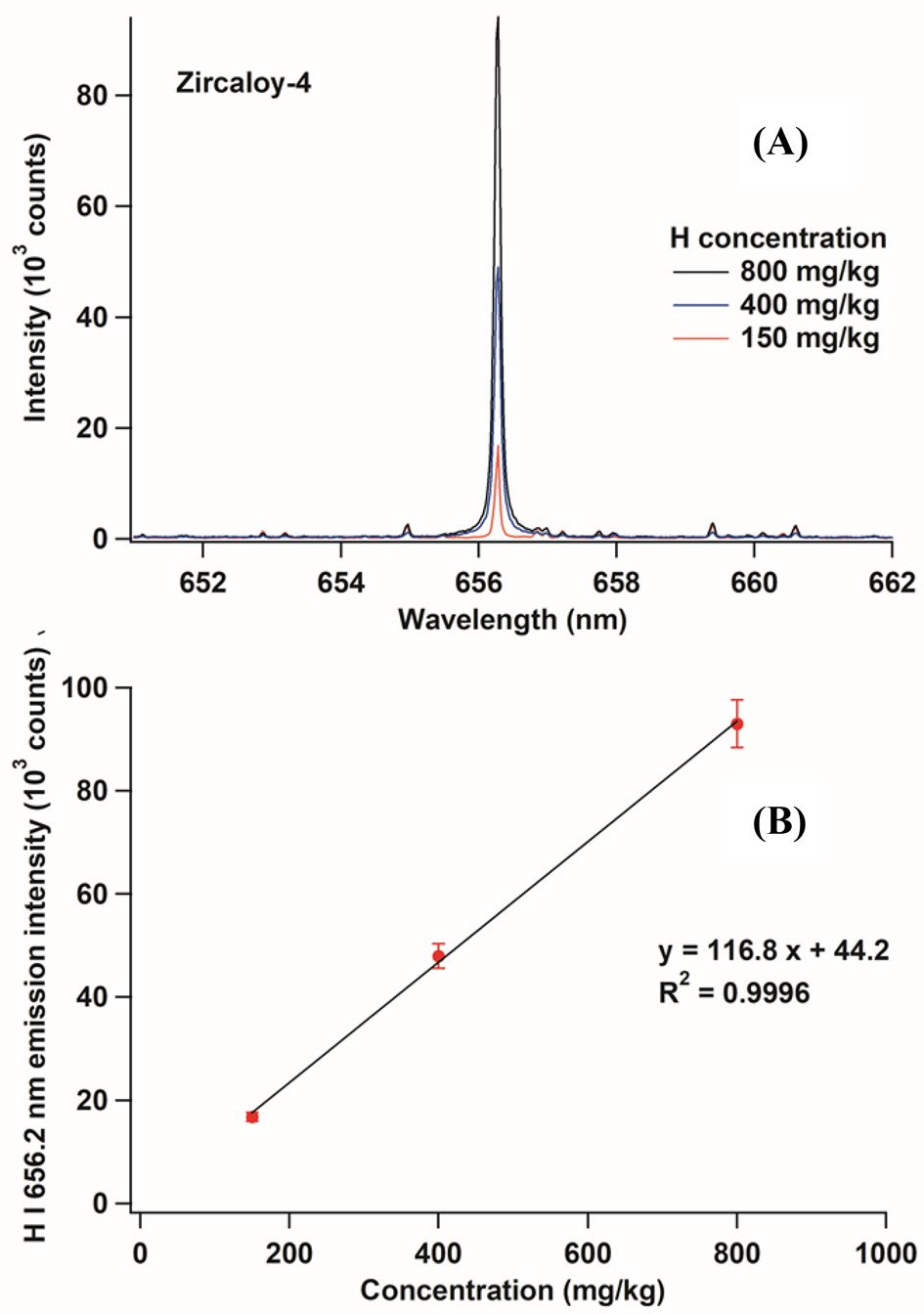
(**A**) Emission spectra of zircaloy-4 sample containing 150 mg/kg, 400 mg/kg, and 800 mg/kg of H. The conditions were the same as those mentioned in Fig. 4. (**B**) Intensity–concentration curve of H emission line derived from (**A**).

In the next part of the experiment, we used zircaloy-4 samples containing 150 mg/kg, 400 mg/kg, and 800 mg/kg of H. The corresponding emission spectra are shown in Fig. 6A. This figure shows the proportionality between the emission intensity and the content of H in the zircaloy-4 samples. The resulting concentration–intensity plot is shown in Fig. 6B. It clearly presents a suitable linear calibration line with an extrapolated near zero intercept. This can be considered as an indication of the absence of any contribution from the surface water, unlike in the commonly H analysis in metals. Furthermore, by employing the following criterion of detection limit^41^,

9 $${\text{Limit}}\,{\text{of}}\,{\text{detection}}\,{\text{(LOD)}} = \frac{3\,\sigma }{S},$$

where $\sigma$ is the average surrounding noise intensity and *S* is the slope of the calibration curve, the detection limit of the proposed technique was estimated to be approximately 0.51 mg/kg, which is considerably better than the required limit of several hundred milligrams per kilogram for a nuclear power station.

## Conclusion

In this study, we showed that high-sensitivity H analysis of zircaloy-4 samples can be performed with a detection limit of 0.51 mg/kg, which is the lowest detection limit ever reported in literature for H analysis on metals. This superior detection limit was obtained by utilizing He* atoms in a Penning-like energy transfer process and using a unique DP orthogonal LIBS system. Furthermore, the obtained H I 656.2 nm emission line exhibited a very narrow linewidth of 0.5 Å, which opens the possibility to detect simultaneously H and D in zircaloy-4 samples, where their wavelength separation is only 1.8 Å. The detection of D in zircaloy-4 is an important issue nowadays following the rapid development of heavy water nuclear power plant.
